# Differential value of brain magnetic resonance imaging in multiple system atrophy cerebellar phenotype and spinocerebellar ataxias

**DOI:** 10.1038/s41598-019-53980-y

**Published:** 2019-11-22

**Authors:** Minkyeong Kim, Jong Hyeon Ahn, Yoonsu Cho, Ji Sun Kim, Jinyoung Youn, Jin Whan Cho

**Affiliations:** 1 Department of Neurology, Samsung Medical Center, Seoul, Korea; 2 Neuroscience Center, Samsung Medical Center, Seoul, Korea; 3 Sungkyunkwan University School of Medicine, Seoul, Korea

**Keywords:** Neurodegeneration, Spinocerebellar ataxia

## Abstract

Clinically differentiating multiple system atrophy cerebellar (MSA-C) phenotype and spinocerebellar ataxias (SCAs) is challenging especially in the early stage. We assessed diagnostic value of brain magnetic resonance imaging (MRI) in differentiating MSA-C and SCAs based at different disease stages (<3, 3–7, and >7 years of disease duration). Overall, 186 patients with probable MSA-C and 117 with genetically confirmed SCAs were included. Hot cross bun (HCB) signs and middle cerebellar peduncle (MCP) hyperintensities were exclusively prevalent in MSA-C compared to SCAs at <3 years (HCB, 44.6% versus 0.9%; MCP hyperintensities, 38.3% versus 0.9%, respectively). Sensitivity, specificity, and positive predictive value (PPV) for HCB signs to differentiate MSA-C from SCAs were 45%, 99%, and 99% and those for MCP hyperintensities were 68%, 99%, and 99%, respectively; considering both HCB signs and MCP hyperintensities, specificity and PPV were 100%. However, the differential value of MRI signs decreased over time. MCP widths were smaller and showed more significant decrease in MSA-C than in SCAs. In conclusion, pontine and MCP changes were exclusively prominent in early stage MSA-C rather than in SCAs. Therefore, we should consider disease duration when interpreting pontine and MCP changes in brain MRIs, which will help better differentiate MSA-C and SCAs.

## Introduction

Ataxia is a clinically heterogeneous group of disorders, in which multiple system atrophy cerebellar (MSA-C) phenotype denotes idiopathic degenerative cerebellar ataxia, while spinocerebellar ataxias (SCAs) represent autosomal dominant cerebellar ataxia^1,2^. Although aetiologies, diagnostic processes, and prognoses are totally different^3,4^, differentiation should be done very carefully, especially in the early stage of disease. Ataxia could be the only sign of both diseases in the early stage, as autonomic dysfunction or parkinsonism appears 2–5 years later in MSA patients with cerebellar ataxia^5–7^. Some patients with SCAs could also manifest autonomic dysfunction or parkinsonism^8,9^. Furthermore, family history could be negative in patients with SCAs^10^.

Brain magnetic resonance imaging (MRI) could be helpful when making a differential diagnosis, as hot cross bun (HCB) signs and hyperintensities in middle cerebellar peduncles (MCPs) are considered characteristic for MSA-C^11–13^. However, recent reports showed that those signs were found in ataxic patients with other etiologies including SCAs^14–16^. HCB signs and MCP changes reflect sequential gliosis and degenerative process of the pons and related afferent fibers^17,18^. As disease progression rates are different in MSA and SCAs^5,7,19^, the effect of disease duration should be considered. To our knowledge, radiologic changes depending on disease duration of MSA and SCAs have not been studied so far.

We hypothesised that pontine and MCP changes would differ in early-stage brain MRIs of patients with MSA-C and SCAs, which would have a diagnostic value in differentiating MSA-C from SCAs. Thus, we explored the prevalence of HCB signs and MCP changes in brain MRIs based on the duration of MSA-C and SCAs.

## Methods

### Patient selection and baseline data collection

This study was approved by the Institutional Review Board (IRB) of Samsung Medical Centre, Seoul, Korea. All methods were performed in accordance with the relevant guidelines and regulations, and a waiver of informed consent was granted by the IRB. Medical records of patients with probable MSA-C and genetically confirmed SCAs from Samsung Medical Centre in Seoul were retrospectively reviewed. From January 2012 to March 2018, 196 MSA-C patients were initially identified; eventually, 186 patients were included based on the second consensus diagnostic criteria of MSA^4^. Patients who showed a static course, lacked autonomic failure as described in the criteria, reported >75 years as age of onset, or presented with other explicable causes of cerebellar ataxia were excluded. Furthermore, for those who manifested initial symptom before 50 years of age, SCA panel tests were performed to exclude the possibility of autosomal dominant cerebellar ataxia. Regarding SCAs, 157 patients with positive genetic tests visited our centre from January 2011 to March 2018. Patients whose MRI findings were not available or inappropriate for assessment were excluded; finally, 117 patients from 84 families with SCAs were included. Baseline characteristics such as sex, age of onset, and disease duration at the time of MRI acquisition were obtained. Disease duration was calculated from the symptom onset based on medical records, when any of the following features manifested: gait disturbance due to imbalance or slowness, dysarthria, hand incoordination, postural dizziness or syncope, urinary incontinence or retention, erectile dysfunction, dizziness unexplained by otologic problems or postural change; decreased visual acuity, parkinsonism and dystonia were considered initial symptoms as well^20,21^.

### MRI acquisition and analysis

Conventional brain MRIs of the patients were retrospectively reviewed. MRIs were grouped according to the duration of SCAs and MSA-C (<3, 3–7, and >7 years of disease duration) with reference to the clinical course of the disease. The median time period from onset to the point of assisted walking was approximately 3 years and that to bedridden state or death was around 7 years in MSA-C patients^5,7^. Two independent movement disorder specialists (M Kim and JW Cho) who were blinded to the clinical information evaluated all MRIs. In cases of discrepancy between grades assessed by the two neurologists, the final grade for analysis was decided by consensus between the two.

All patients were imaged on 3.0 T system. Presence of HCB signs, hyperintensities in MCPs, dorsolateral hyperintensities in the putamen (putaminal rim signs), or putaminal hypointensities were assessed on T2-weighted axial images (3000–6960 ms repetition time, 80–130 ms echo time, and 3.0–5.0 mm thickness). For MCP atrophy, MCP widths were measured by averaging the distances between the superior and inferior borders of MCPs in both sides on T1-weighted sagittal images [250–717 ms repetition time, 2.9–12.5 ms echo time, 4.0–5.0 mm thickness] (Supplementary Fig. S1)^22^. Pontine signals were identified on three categories, namely, no HCB sign or midline hyperintensities (MH), MH, and HCB signs^23^. HCB signs were considered present when both horizontal and vertical lines were observed^24^. MCP signs were marked as absent or present^11–13^. Regarding putaminal abnormalities, high signals alongside the dorsolateral margin of the putamen (putaminal rim signs) and signal intensities lower than those of adjacent globus pallidus (putaminal hypointensities) were considered significant^25^.

### Statistical analysis

Statistical analyses were performed with a commercially available software package (IBM SPSS Statistic version 25, SPSS Inc., Chicago, IL, USA). Age of onset, sex ratio, and disease duration at MRI acquisition were compared between patients with MSA-C and SCAs. For categorical variables, Pearson’s χ2 test was used; for continuous variables, data distribution was first evaluated using Shapiro-Wilk analysis, and independent t-test or Mann-Whitney test was performed as appropriate. P-values of the difference in frequencies of HCB signs and MCP hyperintensities were evaluated using Pearson’s χ2 test. Independent t-test or Mann Whitney test was used to compare MCP widths between two groups, and Wilcoxon rank test was used for comparison between <3 years and 3–7 years of disease duration within each group. Inter-rater reliability for all images between the two readers was assessed with intra-class correlation coefficient (ICC). p-values < 0.05 were considered statistically significant.

In addition, multinomial regression analysis was performed in the initial and second MRIs of MSA-C and SCAs to investigate if there was any correlation between presence of HCB or MH and clinical variables: age of onset, sex, Scale for the Assessment and Rating of Ataxia, or accompanying symptoms such as gait disturbance, dysarthria, autonomic dysfunction, rapid eye movement sleep behaviour disorder, hand incoordination, dizziness, dysphagia, sensory symptoms, and parkinsonism. Bonferroni’s method was applied to p-value and confidence level to correct the increased size of the type I error due to multiple testing.

Baseline characteristics were compared within SCA subgroups (SCA1, SCA2, SCA3, SCA6, SCA7, SCA8 and SCA17) using Fisher’s exact test for categorical variables and one-way analysis of variance for continuous variables.

## Results

### Patient characteristics and MRI distribution

Baseline characteristics of the subjects are described in Table 1. Sex ratios were not different between the two groups. Age of onset was significantly higher in patients with MSA-C than in those with SCAs (t = 12.225, p = 0.000). In both MSA-C and SCA groups, gait disturbance due to imbalance or slowness was the most common initial symptom (Supplementary Table S2 online). Demographic and genetic characteristics depending on SCA subgroups are demonstrated in Supplementary Table S3.

**Table 1:** Baseline characteristics and MRI distribution.

	MSA-C	SCAs	p-value
No. of patients	186	117	
Men: Women	109:77	66:51	0.707
Age of onset (yr)	57.4 ± 7.5	40.4 ± 13.7	t = 12.225, p = 0.000
Disease duration at Initial MR acquisition (n, yr)	186, 1.3 ± 0.8	117, 1.5 ± 0.9	p = 0.273
Disease duration of MR acquisition during 3–7 yr (n, yr)	40, 4.1 ± 0.9	33, 4.9 ± 1.0	p = < 0.001
Disease duration of MR acquisition during ≥7 yr (n, yr)	NA	44, 14.0 ± 7.1	

All patients had initial MRIs within 3 years of disease duration. Follow-up MRIs were performed in 40 MSA-C patients and in 33 SCA patients at 3–7 years of disease duration. No MSA-C patient had follow-up study after 7 years of disease duration, while 44 patients with SCAs took the second MRI during the same period. Initial MRI was performed at similar disease duration (1.3 ± 0.8 versus 1.5 ± 0.9 years, respectively), while the second MRI was performed earlier in MSA-C patients at 3–7 years of disease duration (4.1 ± 0.9 versus 4.9 ± 1.0 years, respectively). At > 7 years of disease duration, MRIs were available only in the SCA group, as MRI was performed at an average of 14.0 ± 7.1 years.

### Radiologic findings in MSA-C and SCA patients

**Figure 1:**
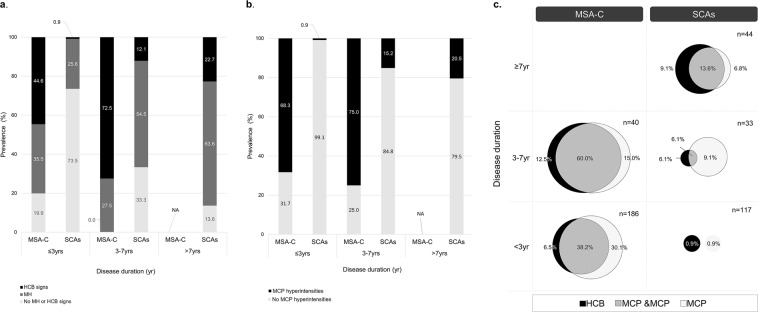
MRI findings in MSA-C and SCAs depending on disease duration. (**a**) HCB signs and (**b**) MCP hyperintensities were more prevalent in MSA-C than in SCAs. (**c**) When HCB signs and MCP hyperintensities were considered together, the differentiation between MSA-C and SCAs became more evident. HCB, hot cross bun; MCP, middle cerebellar peduncle; MSA-C, multiple system atrophy cerebellar phenotype; SCAs, spinocerebellar ataxias.

**Figure 2:**
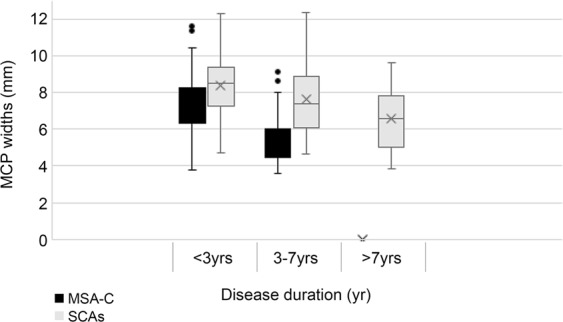
MCP widths in MSA-C and SCAs. MCP widths were smaller in MSA-C than in SCAs at <3 years and 3–7 years of disease duration. The MCP widths for MSA-C and SCA groups significantly decreased overtime when <3 and 3–7 year groups were compared within each group. MCP, middle cerebellar peduncle; MSA-C, multiple system atrophy cerebellar phenotype; SCAs, spinocerebellar ataxias.

The distribution of HCB signs and MCP hyperintensities are illustrated in Fig. 1. At <3 years of disease duration, 83 (44.6%) of 186 MSA-C patients demonstrated HCB signs and 66 (35.5%) showed MH; however, among 117 SCA patients, only one (0.9%) patient with SCA2 manifested definite HCB sign and 30 (25.6%) exhibited MH (Fig. 1a). At 3–7 years of disease duration, 29 (72.5%) MSA-C patients showed HCB signs; 23 (57.5%) patients newly manifested HCB signs and 6 (15.0%) patients had already shown HCB signs in the initial study. MH was observed in 11 (27.5%) patients including 3 (7.5%) patients who had initially shown MH within 3 years of disease duration. Of the 33 SCA patients, HCB signs and MH appeared in four (12.1%) and 18 (54.5%) patients, of which four (12.1%) had already shown MH at <3 years of disease duration. After 7 years of disease duration, 10 (22.7%) SCA patients newly presented HCB signs. MCP hyperintensities were initially detected in 127 (68.3%) of 186 MSA-C patients at <3 years of disease duration, while they appeared only in one (0.9%) of 117 SCA patients (Fig. 1b). Overall, 30 (75.0%) MSA-C patients exhibited MCP hyperintensities at 3–7 years of disease duration; among them, 15 (37.5%) patients newly manifested high signals in MCPs for the first time. In SCAs, MCP hyperintensities newly appeared in five (15.2%) of 33 patients at 3–7 years and in 9 (20.5%) of 44 patients at >7 years of disease duration. Furthermore, when HCB and MCP signs were both considered, the difference between MSA-C and SCAs became more evident (Fig. 1c). The concurrence of HCB signs and MCP hyperintensities was detected in 71 (38.2%) versus 0 (0%) patients at <3 years, and 24 (60.0%) versus 2 (6.1%) patients at 3–7 years of disease duration in MSA-C and SCAs, and the concurrence of both signs in SCA patients at >7 years of disease duration was six (13.6%). T1-weighted sagittal images were available in 124 patients at <3 years and in 38 patients at 3–7 years of disease duration in the MSA-C group and in 51 and 28 patients, respectively, in the SCA group during the same periods. The average MCP width was smaller in MSA-C patients than in SCA patients (7.4 ± 1.5 versus 8.3 ± 1.7 mm, p = 0.000) at <3 years; the difference became more evident (5.4 ± 1.4 versus 7.6 ± 1.8 mm, p = 0.000) at 3–7 years of disease duration (Fig. 2). When the difference between <3 years and 3–7 years of disease duration was compared within each group, MCP widths for MSA-C significantly decreased overtime from 7.2 ± 1.5 to 5.0 ± 1.2 mm (p = 0.000); MCP widths in SCAs also showed marked decrease from 9.2 ± 1.2 to 7.3 ± 1.3 mm (p = 0.001).

Putaminal rim signs and putaminal hypointensities were observed in overall 7 (3.8%) and 12 (6.5%) patients with MSA-C, respectively. In our cohort, no SCA patients exhibited putaminal changes.

### Statistical measures

Sensitivity, specificity, and positive predictive value (PPV) of HCB signs and MCP hyperintensities are shown in Table 2. At <3 years of disease duration, sensitivity of HCB signs to differentiate MSA-C from SCAs was below 50%, while the specificity and PPV were 99.1% and 98.8%, respectively. During the next 3–7 years, sensitivity increased to 72.5%, while specificity and PPV decreased to 87.9%. MCP hyperintensities showed similar pattern. Sensitivity was 68.3%; specificity and PPV were 99.1% and 99.2%, respectively, at <3 years of disease duration, which were then 75.0%, 84.8%, and 85.7% at 3-7 years of disease duration, respectively. When HCB and MCP signs were both considered, the PPV for differentiating MSA-C from SCAs was 100% at <3 years and 92.3% at 3–7 years of disease duration, respectively. Although the p value of difference in MCP widths between MSA-C and SCAs were 0.000 both at <3 years and 3–7 years of disease duration, a large overlap made it unable to set a cut-off value that effectively differentiated the two groups. At 3–7 years of disease duration, sensitivity, specificity and positive predictive value to distinguish MSA-C from SCAs were 76.3%, 78.6% and 82.9% when a cutoff of 6 mm or less was used.

**Table 2:** Statistical measures of MRI findings to differentiate MSA-C from SCAs.

	<3 yr			3–7 yr		
	Sensitivity (%)	Specificity (%)	PPV (%)	Sensitivity (%)	Specificity (%)	PPV (%)
HCB signs	44.6	99.1	98.8	72.5	87.9	87.9
MCP hyperintensities	68.3	99.1	99.2	75.0	84.8	85.7
Coexistence of HCB& MCP signs	38.2	100.00	100.00	60.00	93.9	92.3
MCP width*				76.3	78.6	82.9

*At 3–7 years of disease duration, cutoff of 6 mm or less was used.

The ICC between the two investigators was 0.985 for HCB, 0.977 for MCP, and 0.979 for MCP width. ICCs for putaminal rim signs and putaminal hypointensities were 0.946 and 0.983, respectively.

Multinomial regression analysis revealed that none of the clinical parameters significantly predicted HCB or MH.

### HCB signs and MCP hyperintensities among the SCA subtypes

The overall frequencies of HCB and MCP hyperintensities in SCA patients are described in Table 3. HCB signs and high signals in MCPs were the most prevalent in SCA2, and none of the patients with SCA1 (n = 7), SCA6 (n = 20), and SCA17 (n = 1) manifested those signs.

**Table 3:** Frequencies of HCB and MCP hyperintensities in patients with SCAs.

	SCA1	SCA2	SCA3	SCA6	SCA7	SCA8	SCA17	Sum	P-value
	(n = 7)	(n = 36)	(n = 39)	(n = 20)	(n = 7)	(n = 7)	(n = 1)	(n = 117)	
HCB (n, %)	0	11 (30.6)	2 (5.1)	0	1 (14.3)	1 (14.3)	0	15 (12.8)	<0.001
MCP (n, %)	0	12 (33.3)	1 (2.6)	0	1 (14.3)	1 (14.3)	0	15 (12.8)	0.001

## Discussion

To our knowledge, this is the first study to compare radiologic findings of MSA-C and SCAs according to their disease duration. HCB signs and MCP hyperintensities were exclusively prevalent in MSA-C at <3 years with PPVs of nearly 100%. This value even increased to 100% at the same disease duration when HCB signs and MCP hyperintensities were considered together. As a result, the presence of HCB signs or MCP hyperintensities within 3 years of disease duration would differentiate MSA-C from SCAs. MCP widths were smaller in MSA-C with statistical significance at <3 years as well as at 3–7 years of disease duration, although the differential value of MCP widths was relatively low. Dorsolateral putaminal rim signs and putaminal hypointensities were only detected in the MSA-C group. When only SCAs were considered, HCB signs and MCP hyperintensities were the most frequent in SCA2.

Our results are in line with pathologic findings. A sequential analysis on α-synuclein pathology in MSA-C patients showed that the pontocerebellar and middle cerebellar fibres were initially affected in the pons^26^. While post-mortem analyses in SCAs also revealed various degrees of cerebellar and brainstem involvement, reports regarding early stage pathology are limited^27^. Autopsy specimens from SCA2 and SCA8 patients with 3 years of disease duration demonstrated no involvement of pontocerebellar fibres^28,29^. Furthermore, these radiologic and pathologic findings appear to be related with the clinical course. Functional state of the patients was also assessed in terms of walking ability during MRI acquisition, in which more MSA-C patients required assistance or were wheelchair bound compared to SCA patients (Supplementary Table S4). This result was in accordance with the results of previous studies, wherein MSA-C patients showed more rapid deterioration in activities of daily living compared to those with SCAs^30^. Therefore, early appearance of HCB signs and MCP changes paralleled the pathologic changes involving pontocerebellar fibres, which may be related with clinical findings based on the time course.

The prevalence of putaminal rim signs or hypointensities in this study was relatively lower to that in previous studies^11–13^. The low incidence of putaminal change in this study may have been due to the correlation of parkinsonism with putaminal changes in brain MRIs and that subjects were limited to those with predominant cerebellar phenotype^12^.

The overall frequencies of HCB signs were 12.8% in SCA patients, which was similar to that reported in a Taiwanese study (8.7%) wherein SCA2 was also the most common subtype^15^. Moreover, the similar results in both populations may have been attributed to the high involvement of the pontine nucleus in SCA2 than in any other subtypes^27,31^. However, disease duration was not specified in the Taiwanese report^15^. Other case series reporting HCB signs in SCAs did not take into account disease duration; in fact, MRIs were taken at more than 5 years of disease duration^32–34^. In our SCA cohort, the overall HCB signs appeared at 11.1 ± 6.0 years of disease duration.

For those who had follow-up MRIs, we additionally investigated how the pontine and MCP signals had changed in MSA-C and SCAs (Supplementary Table S5). We observed a common process of HCB formation in both disease groups: vertical lines appeared first, followed by horizontal lines^23,24^. More MSA-C patients eventually came to have HCB signs (67.6% versus 18.2%) while SCA patients turned to have MH without progression to HCB signs. Similar to HCB signs, more MSA-C patients exhibited MCP hyperintensities than SCA patients in follow-up studies (60.0% versus 18.2%).

The most critical limitation of the current study was that pathological study results was not available and SCA panel tests were performed in a limited number of MSA-C patients^4,35^. However, all patients fulfilled the diagnostic criteria of probable MSA^4^. As the age of onset in genetically confirmed SCAs was around 40 years in our cohort, SCA diagnoses were ruled out in MSA-C patients with <50 years of age of onset using SCA panel test. The possibility of SCA6, which is known for late-onset cerebellar ataxia, was discarded as well, since pure cerebellar ataxia distinguished SCA6 from MSA-C. Second, age of onset, which was based on medical records, might have been inaccurate. Gait disturbance was initially the most common chief complaint and was accompanied by various motor or non-motor symptoms in MSA (Supplementary Table S2); however, it was not easy to identify accurately when all those symptoms started. In fact, a retrospective review of questionnaires revealed that postural dizziness and erectile dysfunction were the most prevalent initial symptoms in MSA, which were found in 58.1% and 38.7% of our MSA-C patients at initial visit, respectively; urinary symptoms prevailed among non-motor symptoms^20^. However, as gait disturbance due to postural instability or slowness was detected during the first year of disease onset^20^, we thought that it was possible to approximate disease duration. In the SCA group, gait disturbance was the most common initial symptom as previously described^21^.

We compared radiologic signs between MSA-C and SCAs for the first time, especially focusing on the disease duration. All patients in our cohort underwent MRI within 3 years after symptom onset; however, none of MSA-C patients had an MRI after 7 years, whereas SCA patients had MRI at >10 years of disease duration. This reflects that ambiguous results are often encountered in the early stage of ataxias, while clinical features become more evident after 7 years of disease duration, requiring fewer tests to differentiate MSA-C from SCAs. Obviously, a thorough history taking, neurologic examination, and other diagnostic modalities are necessary, but early HCB signs and MCP hyperintensities have diagnostic value in MSA-C and SCAs; if MCP shows marked atrophy, which also suggests MSA-C rather than SCAs.

In conclusion, we should take into account disease duration when interpreting pontine and MCP changes in brain MRIs, which helps better differentiate MSA-C and SCAs in synopsis with clinics and genetics. This would provide chances not only to anticipate prognosis but also to apply symptomatic and even disease modifying therapies.

## Supplementary information

Supplementary Information

## Data Availability

All data generated or analyzed during this study are included in this published article (and its Supplementary Information Files).

## References

[1] Klockgether T (2010). Sporadic ataxia with adult onset: classification and diagnostic criteria. Lancet Neurol..

[2] Kim JS, Cho JW (2015). Hereditary cerebellar ataxias: A Korean perspective. J Mov Disord..

[3] TD., B. Hereditary ataxia overview. *GeneReviews®*, https://www.ncbi.nlm.nih.gov/books/NBK1138/, (accessed 2019).

[4] Gilman S (2008). Second consensus statement on the diagnosis of multiple system atrophy. Neurology.

[5] Tada M (2007). Early development of autonomic dysfunction may predict poor prognosis in patients with multiple system atrophy. Arch Neurol.

[6] Gilman S (2000). Evolution of sporadic olivopontocerebellar atrophy into multiple system atrophy. Neurology.

[7] Watanabe H (2002). Progression and prognosis in multiple system atrophy: an analysis of 230 Japanese patients. Brain.

[8] De Joanna G (2008). Autonomic nervous system abnormalities in spinocerebellar ataxia type 2: a cardiovascular neurophysiologic study. J Neurol Sci.

[9] Furtado S (2004). Profile of families with parkinsonism-predominant spinocerebellar ataxia type 2 (SCA2). Mov Disord.

[10] Kim JS, Kwon S, Ki CS, Youn J, Cho JW (2018). The etiologies of chronic progressive cerebellar ataxia in a Korean population. J Clin Neurol.

[11] Bürk K (2005). Clinical and magnetic resonance imaging characteristics of sporadic cerebellar ataxia. Arch Neuro.

[12] Lee EA, Cho HI, Kim SS, Lee WY (2004). Comparison of magnetic resonance imaging in subtypes of multiple system atrophy. Parkinsonism Relat Disord.

[13] Brooks DJ, Seppi K (2009). Proposed neuroimaging criteria for the diagnosis of multiple system atrophy. Mov Disord.

[14] Christopher W, David P, Amie H (2019). The ‘Hot Cross Bun’ sign is not always multiple system atrophy: etiologies of 11 Cases. J Mov Disord.

[15] Lee YC (2009). The ‘hot cross bun’ sign in the patients with spinocerebellar ataxia. Eur J Neurol.

[16] Okamoto K (2003). MR features of diseases involving bilateral middle cerebellar peduncles. Am J Neuroradiol.

[17] Takao M, Kadowaki T, Tomita Y, Yoshida Y, Mihara B (2007). ‘Hot-cross bun sign’ of multiple system atrophy. Intern Med.

[18] Morales H, Tomsick T (2015). Middle cerebellar peduncles: Magnetic resonance imaging and pathophysiologic correlate. World J Radiol.

[19] Diallo A (2018). Survival in patients with spinocerebellar ataxia types 1, 2, 3, and 6 (EUROSCA): a longitudinal cohort study. Lancet Neurol.

[20] McKay JH, Cheshire WP (2018). First symptoms in multiple system atrophy. Clin Auton Res.

[21] Luo L (2017). The initial symptom and motor progression in spinocerebellar ataxias. Cerebellum.

[22] Nicoletti G (2006). MR imaging of middle cerebellar peduncle width: differentiation of multiple system atrophy from Parkinson disease. Radiology.

[23] Higashi M (2018). A diagnostic decision tree for adult cerebellar ataxia based on pontine magnetic resonance imaging. J Neurol Sci.

[24] Horimoto Y (2002). Longitudinal MRI study of multiple system atrophy - when do the findings appear, and what is the course?. J Neurol.

[25] Watanabe H (2010). Putaminal magnetic resonance imaging features at various magnetic field strengths in multiple system atrophy. Mov Disord.

[26] Brettschneider J (2017). Progression of alpha-synuclein pathology in multiple system atrophy of the cerebellar type. Neuropathol Appl Neurobiol.

[27] Seidel K (2012). Brain pathology of spinocerebellar ataxias. Acta Neuropathol.

[28] Hoche F (2011). Spinocerebellar ataxia type 2 (SCA2): identification of early brain degeneration in one monozygous twin in the initial disease stage. Cerebellum.

[29] Ito H (2006). Clinicopathologic investigation of a family with expanded SCA8 CTA/CTG repeats. Neurology.

[30] Klockgether T (1998). The natural history of degenerative ataxia: a retrospective study in 466 patients. Brain.

[31] Tada M, Nishizawa M, Onodera O (2015). Redefining cerebellar ataxia in degenerative ataxias: lessons from recent research on cerebellar systems. J Neurol Neurosurg Psychiatry.

[32] Wang Y, Koh K, Takaki R, Shindo K, Takiyama Y (2016). Hot cross bun sign in a late-onset SCA1 patient. Neurol Sci.

[33] Gooneratne IK, Caldera MC, Perera SP, Gamage R (2013). Hot cross bun sign in a patient with cerebellar ataxia. Ann Indian Acad Neurol.

[34] Pedroso JL, Rivero RL, Barsottini OG (2013). “Hot cross bun” sign resembling multiple system atrophy in a patient with Machado-Joseph disease. Arq Neuropsiquiatr.

[35] Kim HJ (2014). Should genetic testing for SCAs be included in the diagnostic workup for MSA?. Neurology.

