# Supplementary material for: Differential value of brain magnetic resonance imaging in multiple system atrophy cerebellar phenotype and spinocerebellar ataxias
Source: Sci Rep. 2019 Nov 22;9:17329. doi: 10.1038/s41598-019-53980-y (PMC6874541; doi:10.1038/s41598-019-53980-y)
Supplement: Supplementary file 1 — Supplementary Information [file 41598_2019_53980_MOESM1_ESM.pdf]

# Differential value of brain magnetic resonance imaging in multiple system atrophy cerebellar phenotype and spinocerebellar ataxias

Minkyeong Kim, MD, Jong Hyeon Ahn, MD, Yoonsu Cho, Ji Sun Kim, MD, PhD, Jinyoung Youn, MD, PhD, Jin Whan Cho, MD, PhD

Supplementary Figure S1. MRI findings

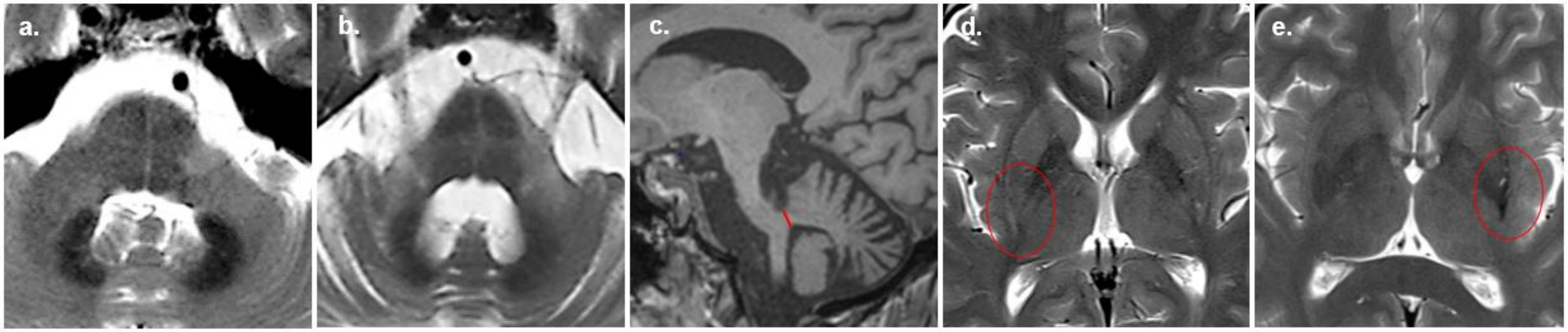


MRI, magnetic resonance imaging; MH, midline hyperintensities; MCP, middle cerebellar peduncle; MSA-C, multiple system atrophy cerebellar phenotype; HCB, hot cross bun

(a) MH and MCP hyperintensities in one MSA-C patient with 0.7 year of disease duration. (b) HCB sign and MCP hyperintensities in another MSA-C patient with 4.4 years of disease duration. (c) MCP width of the same patient as (b). (d) Putaminal rim sign and (d) Putaminal hypointensities are marked with red circles, respectively.

Supplementary Table S2. Symptoms at initial visit

| MSA-C (n, %) | | | | SCAs (n, %) | | | |
|---|---|---|---|---|---|---|---|
| Chief complaint at initial visit | | Overall symptoms at initial visit | | Chief complaint at initial visit | | Overall symptoms at initial visit | |
| Gait disturbance | 125 (67.2) | Gait disturbance | 181 (97.3) | Gait disturbance | 91 (77.8) | Gait disturbance | 111 (94.9) |
| Orthostatic dizziness | 15 (8.1) | Dysarthria | 154 (82.8) | Dysarthria | 12 (10.3) | Dysarthria | 82 (70.1) |
| Dysarthria | 12 (6.5) | Urinary symptom** | 133 (71.5) | Parkinsonism | 5 (4.3) | Dysphagia | 15 (12.8) |
| Dizziness* | 11 (5.9) | RBD | 128 (68.8) | Hand incoordination | 5 (4.3) | Hand incoordination | 12 (10.3) |
| Hand incoordination | 10 (5.4) | Orthostatic dizziness | 108 (58.1) | Decreased visual acuity | 3 (2.6) | Urinary symptom** | 11 (9.4) |
| Urinary symptom** | 9 (4.8) | Erectile dysfunction | 72 (38.7) | Dystonia | 1 (0.9) | Orthostatic dizziness | 6 (5.1) |
| Parkinsonism | 4 (2.2) | Hand incoordination | 34 (18.3) | Cognitive decline | 1 (0.9) | Double vison | 5 (3.4) |
| | | Dizziness* | 14 (7.5) | | | Parkinsonism | 6 (5.1) |
| | | Dysphagia | 10 (5.4) | | | Sensory sx. | 5 (4.3) |
| | | Sensory symptom | 6 (3.2) | | | Dystonia | 4 (3.4) |
| | | Parkinsonism | 4 (2.2) | | | Erectile dysfunction | 4 (3.4) |
| | | | | | | Cognitive decline | 4 (3.4) |
| | | | | | | Dizziness* | 4 (3.4) |
| | | | | | | Decreased visual acuity | 3 (2.6) |
| | | | | | | Restless leg syndrome | 1 (0.9) |
| | | | | | | Cramps | 1 (0.9) |

MSA-C, multiple system atrophy cerebellar phenotype; SCAs, spinocerebellar ataxias; RBD, rapid eye movement sleep behaviour disorder

* dizziness unexplained by otologic problems or postural change

** urinary incontinence or retention

Supplementary Table S3. Demographic and genetic characteristics of SCAs

| | SCA1 | SCA2 | SCA3 | SCA6 | SCA7 | SCA8 | SCA17 | **p** |
|---|---|---|---|---|---|---|---|---|
| No. of patients (n, %) | 7 (6.0) | 36 (30.8) | 39 (33.3) | 20 (17.1) | 7 (6.0) | 7 (6.0) | 1 (0.9) | |
| Men: Women | 4: 3 | 23: 13 | 20: 19 | 12: 8 | 3: 4 | 4: 3 | 0: 1 | 0.868 |
| Age of onset (yrs) | 43.7 ± 7.1 | 37.9 ± 12.5 | 39.9 ± 12.9 | 45.7 ± 15.2 | 34.3 ± 23.4 | 43.4 ± 12.3 | 43.5 | F=1.266, p=0.284 |
| TNR No. | 51.4 ± 20.4 | 40.6 ± 4.2 | 68.9 ± 7.0 | 23.0 ± 1.8 | 42.7 ± 7.8 | 100.7 ± 19.6 | 42 | |

SCAs, spinocerebellar ataxias; TNR, trinucleotide repeat

Supplementary Table S4. Walking abilities at the time of MRI acquisition

| | | Independent walking | Require assistance | Wheelchair bound | Bedridden | p-value |
|---|---|---|---|---|---|---|
| **< 3 yr** | MSA-C (n=186) | 150 (80.6) | 32 (17.2) | 4 (2.2) | 0 | 0.000 |
| | SCAs (n-117) | 115 (98.3) | 2 (1.7) | 0 (0.0) | 0 | |
| **3-7 yr** | MSA-C (n=40) | 8 (20.0) | 20 (50.0) | 7 (17.5) | 5 (12.5) | 0.000 |
| | SCAs (n=33) | 25 (75.8) | 7 (21.2) | 1 (3.0) | 0 | |
| **≥ 7 yr** | MSA-C (n=0) | 0 | 0 | 0 | 0 | NA |
| | SCAs (n=44) | 18 (40.9) | 23 (52.3) | 3 (6.8) | 0 | |

MRI, magnetic resonance imaging; MSA-C, multiple system atrophy cerebellar phenotype; SCAs, spinocerebellar ataxias

Supplementary Table 5. Signal changes in the pons and MCPs in follow-up studies*

A. Pontine signal changes

| Initial | | | follow up | | |
|---|---|---|---|---|---|
| | | | no MH or HCB | MH | HCB |
| MSA-C | no MH or HCB | 21 | 0 | 8 | 13 |
| | MH | 13 | 0 | 3 | 10 |
| | sum | 34 | 0 | 11 (32.4%) | 23 (67.6%) |
| SCAs | no MH or HCB | 68 | 21 | 34 | 13 |
| | MH | 9 | 0 | 8 | 1 |
| | sum | 77 | 21 (27.3%) | 42 (54.5%) | 14 (18.2%) |

B. MCP signal changes

| Initial | | | follow up | |
|---|---|---|---|---|
| | | | absent | Present |
| MSA-C | Absent | 25 | 10 (40.0%) | 15 (60.0%) |
| SCAs | Absent | 77 | 63 (81.8%) | 14 (18.2%) |

MSA-C, multiple system atrophy cerebellar phenotype; SCAs, spinocerebellar ataxias; MH, midline hyperintensity; HCB, hot cross bun; MCP, middle cerebellar peduncle

* Follow-up studies were available in 40 and 77 patients with MSA-C and SCAs. Those who already had shown HCB signs (6 patients) or MCP hyperintensities (15 patients) in MSA-C group during <3 years were excluded in this analysis.
